# Mutational Landscape of *FGFR4* Across Malignancies: A Cross-Cancer Analysis of the AACR Project GENIE Database

**DOI:** 10.3390/cimb48070748

**Published:** 2026-07-22

**Authors:** Henna Ali, Tyler Gengnagel, Salem Birkholz, Gowri Vadmal, Elijah Torbenson, Beau Hsia, Abubakar Tauseef, Peter T. Silberstein

**Affiliations:** 1 School of Medicine, Creighton University, Omaha, NE 68178, USA; hal44158@creighton.edu (H.A.); tylergengnagel@creighton.edu (T.G.); salembirkholz@creighton.edu (S.B.); gowrivadmal@creighton.edu (G.V.); elijahtorbenson@creighton.edu (E.T.); abubakartauseef@creighton.edu (A.T.); 2 School of Medicine, Creighton University, Phoenix, AZ 85012, USA; beauhsia@creighton.edu; 3 Department of Hematology/Oncology, CHI Henry Lynch Cancer Center, Omaha, NE 68124, USA

**Keywords:** FGFR4, fibroblast growth factor receptor, pan-cancer analysis, AACR Project GENIE, somatic mutation, tumor mutational burden, receptor tyrosine kinase, precision oncology

## Abstract

Background/Aim: *Fibroblast growth factor receptor 4 (FGFR4)* is a tyrosine kinase involved in cell growth, proliferation, and angiogenesis. While FGFR1–3 are well studied in cancer, FGFR4 remains relatively understudied, and the distribution of its mutations across cancers and patient populations is not well defined. Materials and Methods: A retrospective pan-cancer analysis was performed using the AACR Project GENIE v12 database via cBioPortal. Tumors with somatic *FGFR4* mutations were included, excluding copy number alterations and structural variants. Mutations were grouped by hotspot (amino acid 401) and major protein domains. Comparative analyses assessed cancer type distribution, demographics, mutation burden, and co-occurring genomic alterations using chi-square testing with multiple comparison correction. Results: A total of 4565 tumor samples (4283 patients) were analyzed. *FGFR4* alterations were observed across diverse malignancies, most commonly non-small cell lung cancer, colorectal cancer, and melanoma. Mutations clustered primarily in the tyrosine kinase and immunoglobulin I-set domains, with no significant variation in distribution across cancer types. Sex was not associated with the mutation group, while race and ethnicity showed significant differences. The FGFR4 hotspot 401 group demonstrated a higher mutation burden, driven by a subset of hypermutated tumors, and showed enrichment for co-occurring alterations in chromatin remodeling, DNA repair, tumor suppressor, and receptor tyrosine kinase genes; however, sensitivity analyses indicated this association was largely attributable to mutation burden rather than a mutation-specific effect. Domain-based mutation groups had lower mutation burdens and fewer co-alterations. Conclusions: FGFR4 alterations occur across a broad range of cancers with consistent domain-level patterns. The hotspot 401 mutation shows a higher mutation burden and co-alteration frequency driven largely by a subset of hypermutated tumors, rather than acting as an isolated driver.

## 1. Introduction

Fibroblast growth factor receptor 4 (FGFR4) is a member of the FGFR tyrosine kinase family [1,2]. FGFRs are well-established in research with their physiological roles including cell growth, proliferation, and angiogenesis [1,2]. Overactivation of the FGFR family, particularly FGFR1-3, has been implicated in the growth and progression of many cancers, such as breast, liver, lung, gastric, uterine, bladder, and rhabdomyosarcomas [3].

Similar to FGFR1-3, *FGFR4* is implicated in the progression of many oncologic processes.

What distinguishes *FGFR4* from the rest of the members is that it has the least homology among the FGFR family [4]. In addition, *FGFR4* has a lower gene mutation burden, making the pro-oncogenic gene variations more stable and similar relative to the variety in the other FGFR members [1]. The relative similarity of aberrant *FGFR4* overexpression suggests the development of drug therapy targeting a wide range of cancers.

Unlike the other members of the FGFR family in relation to cancers, *FGFR4* is relatively understudied [1]. Most research on the gene has been conducted to investigate *FGFR4*’s relationship with hepatocellular carcinoma and breast cancer [1,5]. A more comprehensive list includes: lung adenocarcinoma, breast cancer, squamous cell carcinoma of the head and neck, gastric cancer, hepatocellular carcinoma, urothelial carcinoma, colorectal carcinoma, prostate cancer, rhabdomyosarcoma, and ovarian cancer [6]. These lists of *FGFR4* overexpression in cancers primarily look into the type of mutation indicated in certain cancers [6]. What remains unclear is which cancers are most commonly associated with specific *FGFR4* mutations, as well as the patient populations in which these mutations occur most frequently.

Additionally, while several recurrent FGFR4 hotspot mutations have been identified and investigated as potential therapeutic targets, most FGFR4 alterations remain poorly characterized. Existing studies have largely focused on individual mutations within specific cancer types, leaving the broader distribution, co-occurring genomic landscape, and potential biological significance of FGFR4 alterations across diverse malignancies and patient populations incompletely understood [7].

This study aims to determine the frequency of cancers and patient populations associated with certain *FGFR4* mutations. This was accomplished by using the AACR Project GENIE database to map mutation frequencies, demographic and molecular subtype differences, and patterns of genetic co-occurrence or exclusivity. This characterization may help guide the development of molecularly tailored therapies, enhance prognostic assessment, and inform future treatment guidelines.

## 2. Methods

This was a retrospective pan-cancer study of tumors with somatic *FGFR4* alterations using the AACR Project GENIE v.12 database through cBioPortal. The initial search included samples with *FGFR4* mutations. Structural variants and copy number alterations were excluded so that the analysis remained limited to small sequence-level alterations. Using these criteria, the final study cohort included 4565 tumor samples from 4283 patients.

*FGFR4* alterations were grouped according to hotspot position or annotated protein domain on the cBioPortal lollipop plot. Initial groups included hotspot residues 10, 136, 388, and 401, as well as the immunoglobulin I-set domains spanning amino acids 161–236 and 256–350 and the protein tyrosine kinase domain spanning amino acids 467–743. Because hotspots at amino acids 10, 136, and 388 were represented by very few samples, they were excluded from downstream comparative analyses. Subsequent group-based analyses were therefore limited to hotspot 401 and the three major structural regions. The new total number of patients was 2631 and the new total number of samples was 2800. Samples were assigned to non-overlapping groups using the cBioPortal comparison workflow. Mutations were assigned to groups based solely on their amino acid position. A mutation was included in a domain group if its position fell within the annotated amino acid range of that domain as defined on the cBioPortal lollipop plot. Mutations falling outside the amino acid range of any annotated domain or hotspot were excluded from group-based comparative analyses. No computational clustering algorithms were applied.

Comparative analyses were performed in cBioPortal across the retained *FGFR4* mutation groups, consisting of hotspot 401 and the three annotated protein domains, using non-overlapping samples and patients. We compared the distribution of these groups across cancer types and available clinicopathologic variables, including sex, race, ethnicity, and sample type. We also assessed patterns of co-occurring and mutually exclusive genomic alterations to define the broader molecular context of each mutation group, and the most relevant recurrent alterations were summarized in the corresponding figure and tables.

Categorical variables were compared using the chi-square test, with multiple testing corrections performed by the Benjamini–Hochberg method. A *p*-value < 0.05 and a q-value < 0.05 were considered statistically significant. Statistical outputs were taken directly from the cBioPortal comparison module. To assess whether observed co-occurring alteration frequencies were confounded by cancer type or mutation burden, we performed additional sample-level sensitivity analyses using binary gene-level mutation status. These analyses restricted comparisons to shared cancer types, applied cancer-type-stratified Mantel–Haenszel testing, and used multivariable adjustment for mutation count, cancer type, sample type, and sequencing panel.

Driver versus passenger mutations were not inferred de novo in this study but were based on curated annotations provided within the cBioPortal/GENIE framework. Variant classification was determined using the OncoKB precision oncology knowledge base, which integrates biological, clinical, and functional evidence to categorize mutations as oncogenic, likely oncogenic, or variants of unknown significance (VUS) [7]. Recurrent hotspot mutations were identified using large-scale cohort data, where clustering of mutations at specific amino acid residues suggests positive selection and functional importance [8]. Variants lacking recurrence or functional annotation were classified as VUS. Therefore, mutations labeled as “drivers” in this study reflect previously established or strongly supported functional variants, whereas VUS alterations lack sufficient evidence of oncogenic activity.

## 3. Results

### 3.1. Patient Cohort Demographics

The AACR project GENIE database contained 4565 tumor samples and 4283 patients with a somatic *FGFR4* mutation, excluding structural variants and copy number alterations (CNA). The median age of the cohort was 64 years. A total of 51.1% of patients were female, and 46% were male.

### 3.2. Oncologic Distribution of FGFR4 Alterations Across Malignancies

**Figure 1:**
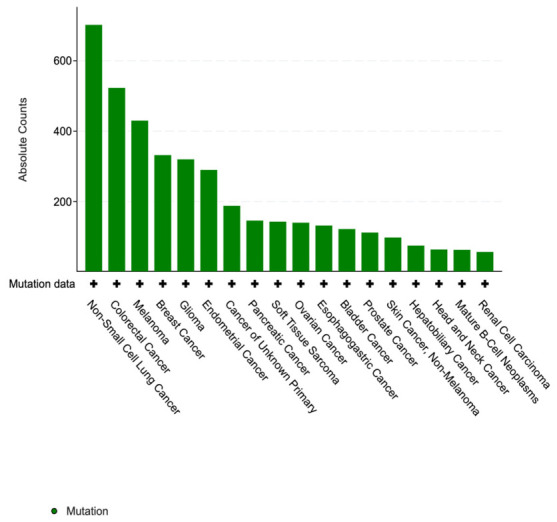
Distribution of FGFR4 mutations across cancer types.

Oncogenic *FGFR4* alterations were observed across a wide range of malignancies seen in Figure 1. The most prevalent cancer types in our cohort included non-small cell lung cancer (2.61%, *n* = 702), colorectal cancer (2.98%, *n* = 523), and melanoma (6.25%, *n* = 430). Breast cancer (2.1%, *n* = 332), glioma (2.45%, *n* = 320), and endometrial cancer (3.73%, *n* = 290) also represented a significant proportion of the cohort.

### 3.3. Patterns of Domain and Hotspot Involvement in FGFR4 Alterations Across Malignancies

**Figure 2:**
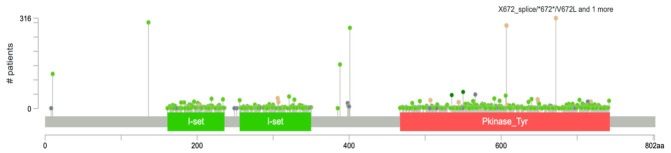
Distribution of *FGFR4* mutations along the protein sequence. Mutations cluster predominantly within the Immunoglobulin I-set domains and the tyrosine kinase domain, with hotspot mutations.

**Figure 3:**
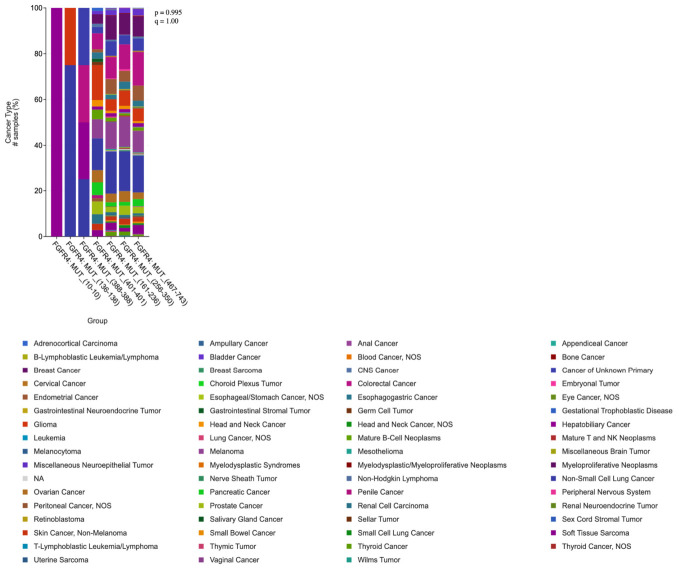
Proportional distribution of cancer types across different mutation-based groups.

Mutations were clustered in several key functional domains. The immunoglobulin I-set domain (256–350) and protein tyrosine kinase (467–743) were the most frequent sites of alteration, accounting for 15.13% and 54.11%, respectively. Another notable domain included immunoglobulin I-set domain (161–236) which occurred at a smaller frequency of 14.1%. Hot spot mutations at amino acids 10, 136, 388, and 401 were also noted at smaller occurrences at 0.036%, 0.14%, 0.14%, and 2.56%, respectively. These hotspots and domains are shown in Figure 2. Hotspots at amino acids 10 (*n* = 1), 136 (*n* = 4), and 388 (*n* = 4) were excluded from further analysis due to limited sample size, whereas the hotspot at amino acid 401 (*n* = 72) was retained. The new total number of patients was 2631 and the new total number of samples was 2800. The full distribution of cancer types across all mutation groups, including excluded hotspots, is provided in Figure 3 and Supplementary Table S1.

The distribution of cancer types across these domains and hot spots did not differ significantly (Chi-square test, *p* = 0.995; *q* = 1.00), suggesting a broadly consistent pattern of domain and hotspot involvement across samples. Across the different domains and hotspots shown in Figure 3, cancer types vary in their relative frequencies, but a few patterns stand out. Non-small cell lung cancer emerges as the most common cancer across all domains, accounting for 18.69% of samples in *FGFR4*: MUT (161–236), 17.65% in *FGFR4*: MUT (256–350) and 16.58% in *FGFR4*: MUT (467–743), consistently representing the largest proportion of samples in each group. Other relatively frequent cancers include melanoma, breast cancer, colorectal cancer, and various hematologic malignancies such as B-cell leukemias/lymphomas, though their proportions fluctuate depending on the specific mutation group.

### 3.4. Demographic Variables Across FGFR4 Mutation Groups

**Figure 4:**
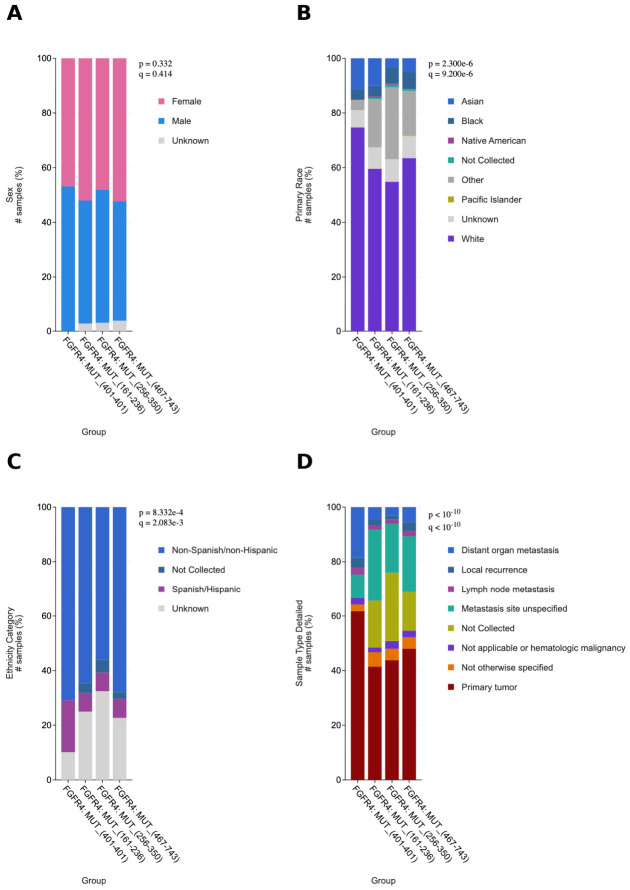
Demographic and sample distribution across *FGFR4* mutation groups. (**A**) Sex distribution. (**B**) Race distribution. (**C**) Ethnic distribution. (**D**) Sample distribution.

The distribution of sex across *FGFR4* mutation groups showed a relatively balanced representation between females and males as shown in Figure 4, with no statistically significant differences (*p* = 0.332, q = 0.414). In the *FGFR4* MUT 401 group, males were slightly more prevalent (53.16%) compared to females (46.84%). In contrast, the *FGFR4* (161–236) and *FGFR4* (467–743) groups demonstrated a modest female predominance, with females comprising 52.02% and 52.23% of samples, respectively, compared to 45.2% and 43.96% in males. The *FGFR4* (256–350) group showed nearly equal representation between sexes (48% females vs. 48.94% males). Overall, these findings indicate that *FGFR4* mutation distribution does not exhibit a strong sex bias across the analyzed cohorts, suggesting that sex is unlikely to be a major confounding factor in downstream analyses of FGFR4-associated outcomes.

The distribution of primary race across *FGFR4* mutation groups revealed statistically significant differences (*p* < 0.001) as shown in Figure 4B. White patients constituted the majority across all groups, though their representation varied, ranging from 74.68% in the *FGFR4* MUT (401) group to 54.82% in the *FGFR4* MUT (256–350) group. Asian patients were represented at varying proportions across groups, comprising 11.39% of the FGFR4 MUT (401) group and 10.10% of the FGFR4 MUT (161–236) group, compared to 3.29% in the FGFR4 MUT (256–350) group and 5.03% in the FGFR4 MUT (467–743) group. The “Other” racial category also showed notable variation, comprising 26.35% of the *FGFR4* MUT (256–350) group compared to only 3.80% of the *FGFR4* MUT (401) group. Black patients represented a relatively consistent minority across all groups, ranging from 3.79% to 6.23%.

The distribution of ethnicity across *FGFR4* mutation groups also demonstrated statistically significant differences (*p* = 8.332 × 10^−4^, q = 2.083 × 10^−3^) shown in Figure 4C. Non-Spanish/non-Hispanic patients comprised the majority in all groups, ranging from 56.00% in the *FGFR4* MUT (256–350) group to 70.89% in the *FGFR4* MUT (401) group. Spanish/Hispanic patients comprised 18.99% of the FGFR4 MUT (401) group, compared to a range of 6.82% to 6.93% across the other mutation groups.

### 3.5. Sample Type and Tumor Site Distribution Across FGFR4 Mutation Groups

The distribution of sample types across *FGFR4* mutation groups revealed highly significant differences (*p* < 0.001, q < 0.001) shown in Figure 4D. Primary tumors constituted the largest category across all groups but were most prominently represented in the *FGFR4* MUT (401) group (61.73%) compared to the *FGFR4* MUT (161–236) (41.41%), *FGFR4* MUT (256–350) (43.76%), and *FGFR4* MUT (467–743) (48.04%) groups. The *FGFR4* MUT (401) group also had the highest proportion of distant organ metastasis samples (18.52%) relative to the other groups, which ranged from 3.06% to 5.57%. Metastasis site-unspecified samples were more evenly distributed, ranging from 8.64% in the *FGFR4* MUT (401) group to 26.01% in the *FGFR4* MUT (161–236) group. Local recurrence, lymph node metastasis, and hematologic malignancy samples were represented at low frequencies across all groups.

### 3.6. Mutation Count Across FGFR4 Mutation Groups

**Figure 5:**
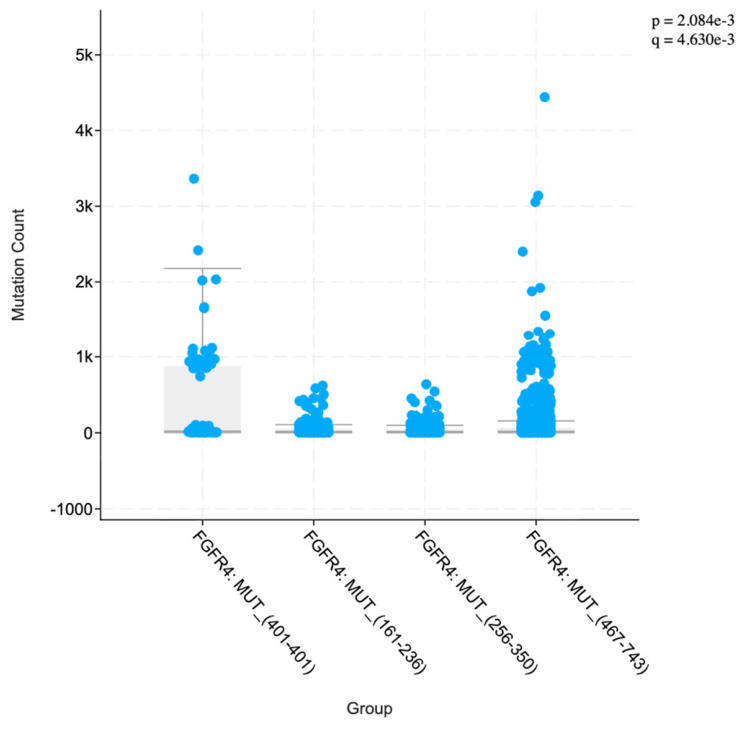
Boxplot of mutation counts across *FGFR4* mutation groups.

The distribution of mutation counts across *FGFR4* mutation groups showed statistically significant differences (*p* = 2.084 × 10^−3^, q = 4.630 × 10^−3^) shown in Figure 5. The *FGFR4* MUT (401) group had the highest mean mutation count (398.43) and the greatest variability (SD = 659.68), with a median of 22 and a range of 2 to 3363. In contrast, the *FGFR4* MUT (161–236) and *FGFR4* MUT (256–350) groups had substantially lower mean mutation counts of 45.67 (SD = 80.36) and 42.04 (SD = 71.1), respectively, with comparable medians of 17 and 16. The *FGFR4* MUT (467–743) group demonstrated an intermediate mean mutation count of 97.4 (SD = 264.65) with a median of 19, though it contained the highest individual outlier in the cohort (4441). These results are summarized in Supplementary Table S2. Across all groups, the mean mutation counts were substantially higher than the median values, indicating that most samples had relatively low mutation counts while a small number of highly mutated samples drove the averages up, particularly in the *FGFR4* MUT (401) and *FGFR4* MUT (467–743) groups.

### 3.7. Co-Occurring Genomic Alterations Across FGFR4 Mutation Groups

To characterize the broader genomic context of *FGFR4* domain-specific mutations, we analyzed patterns of co-occurring alterations across mutation groups. Several genes were enriched for co-occurrence with *FGFR4* mutations, with the *FGFR4* MUT (401) group consistently demonstrating the highest frequency of co-alterations across all genes analyzed. These results are shown in Supplementary Table S3. Tumors harboring the FGFR4 MUT (401) alteration showed a higher observed frequency of co-occurring mutations in chromatin remodeling genes, including ARID1A, CREBBP, and KMT2C, compared to the domain-based groups. Tumor suppressor genes were also frequently co-altered in this group, including APC, NF1, and ATM. DNA damage repair genes BRCA2 and POLE were also more frequently co-altered in the MUT (401) group relative to the domain-based groups. Additionally, Notch pathway genes NOTCH1 and NOTCH3, as well as receptor tyrosine kinase genes ERBB4, ALK, and ROS1, were more frequently co-altered in the FGFR4 MUT (401) group compared to the domain-based groups. In contrast, the domain-based groups showed lower and more comparable frequencies of co-alterations across these same genes.

To evaluate whether these differences were confounded by cancer type or mutation burden, a sample-level sensitivity analysis was performed using binary gene-level mutation status. After excluding samples with mutations in overlapping FGFR4 regions, the MUT(401) group included 47 samples and the comparator group included 2340 samples. All selected co-occurring genes remained more frequent in the MUT(401) group in analyses restricted to shared cancer types and in cancer-type-stratified Mantel–Haenszel testing. However, the MUT(401) group had substantially higher mutation burden, and no co-occurring gene remained significant after excluding hypermutated outliers or after multivariable adjustment for mutation count, cancer type, sample type, and sequencing panel.

### 3.8. Driver vs. Passenger Status of FGFR4 Mutations

**Figure 6:**
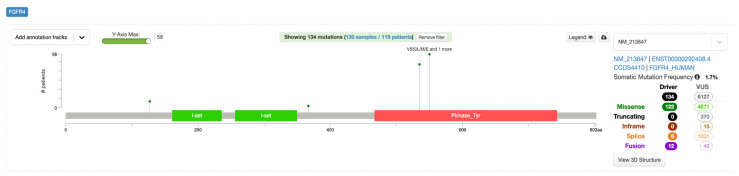
Lollipop diagram of confirmed Driver Mutations along the Protein Sequence.

Of the FGFR4 mutations identified in the AACR GENIE database, only 134 were classified as driver mutations, while 6127 were classified as variants of unknown significance (VUS). The confirmed driver mutations were predominantly missense mutations (*n* = 122), with the remaining classified as fusions (*n* = 12). Driver mutations clustered at two specific residues within the protein tyrosine kinase domain: N535K/D and V550L/M/E/G represented in Supplementary Table S4. The N535K variant was the most frequently observed driver mutation, appearing across multiple cancer types including invasive breast carcinoma and embryonal rhabdomyosarcoma. No truncating, inframe, or splice mutations were classified as drivers. The overall somatic mutation frequency of FGFR4 was 1.7%. This is shown in Figure 6.

### 3.9. Validation Analysis

To validate our findings, we examined the TCGA PanCancer Atlas cohort for *FGFR4* mutations; however, the cohort contained only 85 samples with FGFR4 alterations, which was insufficient to reliably replicate results.

## 4. Discussion

### 4.1. Oncologic Distribution of FGFR4 Alterations Across Malignancies

*FGFR4* alterations in our cohort were distributed across a broad range of malignancies, with the highest representation observed in non-small cell lung cancer, colorectal, and melanoma, followed by endometrial cancer, glioma, and breast cancer, supporting a pan-cancer pattern of *FGFR4* involvement. This is consistent with prior large-scale genomic profiling studies, which have demonstrated that FGFR pathway alterations, while less frequent than other oncogenic drivers, recur across diverse tumor types rather than being confined to a single lineage [9,10,11]. *FGFR4* has historically been less well characterized than FGFR1–3; however, emerging evidence indicates that both activating mutations and overexpression of *FGFR4* contribute to tumor progression, metastatic potential, and therapy resistance in epithelial malignancies such as colorectal and breast cancer, as well as in subsets of hepatocellular carcinoma [1,6,9,10]. Our observation of the relatively high proportion of FGFR4-altered melanoma cases in this cohort adds to the descriptive literature on FGFR4 distribution across various tumor types [12,13]. The presence of *FGFR4* alterations in NSCLC, glioma, and endometrial cancer further supports the biological relevance of *FGFR4* across both epithelial and neuroectodermal-derived tumors, consistent with its role in regulating MAPK and PI3K/AKT signaling pathways implicated in proliferation and survival [1,14,15]. Overall, these findings reinforce FGFR4 as a recurrently altered gene across diverse cancer types and support its continued evaluation as a therapeutic target across multiple tumor types, particularly in the context of emerging selective *FGFR4* inhibitors.

### 4.2. Patterns of Domain and Hotspot Involvement in FGFR4 Alterations Across Malignancies

The mutational landscape of *FGFR4* in our cohort demonstrated alterations within functionally critical domains, with the protein tyrosine kinase domain accounting for the majority of events (54.11%) and the immunoglobulin I-set domains collectively representing a substantial fraction of extracellular alterations.

This pattern is consistent with what is generally known about receptor tyrosine kinases, where activating mutations often occur in the kinase domain and can lead to increased signaling through pathways such as MAPK and PI3K/AKT. In contrast, extracellular immunoglobulin-like domains are more involved in ligand binding and receptor dimerization [16,17,18]. Similar domain-level clustering has been described in FGFR1–3 alterations across cancers, where kinase domain mutations are strongly associated with gain-of-function signaling and oncogenic transformation [6,9]. The presence of recurrent, low-frequency hotspot alterations—including residue 388, which has been previously linked to the *FGFR4* p.Gly388Arg polymorphism—suggests that specific *FGFR4* residues may play a role in influencing receptor activity and tumor behavior [12]. Importantly, the lack of statistically significant differences in domain and hotspot distribution (*p* = 0.995) suggests a broadly conserved pattern of *FGFR4* alteration across tumor types, regardless of cancer lineage.

Prior studies have largely focused on *FGFR4* alterations in single tumor types or emphasized well-known variants such as p.Gly388Arg; however, our study provides a broader, pan-cancer view of *FGFR4* mutational patterns across multiple malignancies. We further extend existing literature by performing a domain-level analysis of *FGFR4* alterations, demonstrating a consistent enrichment within the protein tyrosine kinase domain and a conserved distribution of mutations across tumor types without significant variation in hotspot or domain involvement.

In addition, we identified a low-frequency alteration at residue 401, which has not been well characterized in previous *FGFR4* hotspot analyses and may represent a rare or under-described variant site. Residue 401 is located at the junction of the transmembrane and intracellular juxtamembrane regions of *FGFR4* [19]. The juxtamembrane domain plays an important role in regulating receptor activation, and mutations in this region of receptor tyrosine kinases have been associated with ligand-independent signaling and constitutive activation [20]. Although the functional significance of *FGFR4* residue 401 alterations remains unknown, its location within this regulatory region warrants further investigation. To our knowledge, residue 401 has not previously been identified as a recurrent *FGFR4* hotspot in large pan-cancer studies.

**Figure 7:**
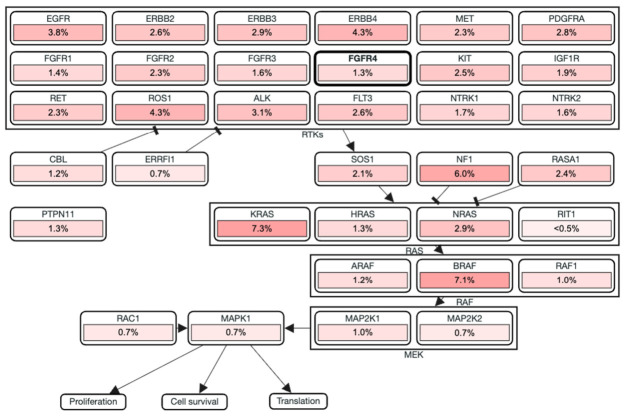
RTK-Ras pathway of FGFR4 and co-altered genes in the retained comparison cohort (*n* = 2631 patients; 2800 samples).

To place FGFR4 alterations within a defined signaling context, we generated an RTK-RAS pathway map using PathwayMapper in cBioPortal for the retained comparison cohort (*n* = 2631 patients; 2800 samples) [21]. PathwayMapper overlays sample-level alteration frequencies onto curated, literature-derived pathway diagrams, allowing genes within a shared signaling cascade to be visualized alongside one another rather than assessed in isolation [21]. Within this pathway shown in Figure 7, FGFR4 was altered in 1.3% of samples, a modest frequency relative to other receptor tyrosine kinases feeding into the same cascade, including ERBB4 (4.3%), ROS1 (4.3%), ALK (3.1%), and EGFR (3.8%). Downstream, KRAS (7.3%) and BRAF (7.1%) showed the highest alteration frequencies in the entire pathway, while NF1 (6.0%), a negative regulator of RAS signaling, was also frequently altered. This distribution illustrates that FGFR4 alterations occur within a broader signaling network in which downstream RAS-MAPK pathway components are altered more frequently than FGFR4 itself. As in our primary analysis, driver versus passenger status for genes depicted in this pathway was based on cBioPortal/OncoKB annotations rather than inferred de novo [7].

Together, these findings offer a more structured, cross-cancer characterization of *FGFR4* and suggest a broadly consistent mutational architecture that has not been previously well described.

### 4.3. Demographic Variables Across FGFR4 Mutation Groups

Across *FGFR4* mutation groups, we observed a relatively balanced distribution between males and females, with no statistically significant differences in sex representation (*p* = 0.332, q = 0.414). Although there were minor variations across domains, these differences were not consistent or large enough to suggest a meaningful sex-based predilection. Notably, sex-based differences in *FGFR4* mutation patterns have not been well characterized in prior genomic studies, as most large-scale analyses of FGFR alterations have focused primarily on tumor type distribution and overall mutation frequency rather than sex-specific stratification. This lack of prior data, together with our findings, suggests that sex is unlikely to be a major determinant of FGFR4 mutation group distribution in this cohort, though further studies would be needed to draw conclusions about underlying biological mechanisms [16,18,22].

The statistically significant differences in racial distribution across FGFR4 mutation groups are noted descriptively. These differences most likely reflect the demographic composition of the institutions contributing to the AACR GENIE consortium rather than true biological variation in FGFR4 mutation frequency across populations. The differences in Asian patient representation across mutation groups are noted, though these findings should be interpreted with caution given the incomplete demographic annotation in the AACR GENIE database. Whether these differences reflect any true population-level variation in FGFR4 mutation patterns cannot be determined from these data.

White patients constituted the majority across all groups, which likely reflects the demographic composition of the institutions contributing to the AACR GENIE consortium rather than a biologically higher susceptibility to *FGFR4* mutations in this population [23,24]. The observed difference in Spanish/Hispanic patient representation in the FGFR4 MUT (401) group is noted descriptively. Given the small sample size of this hotspot group and the limitations of demographic annotation in this registry, no meaningful conclusions can be drawn from this finding. The substantial proportion of samples categorized as “Unknown,” “Other,” or “Not Collected” in both race and ethnicity data represents an important limitation, as incomplete demographic annotation reduces statistical power and may obscure meaningful population-specific patterns. These gaps are a recognized challenge in large-scale genomic databases, as prior analyses have demonstrated that racial and ethnic minorities are underrepresented in major precision oncology registries, including AACR Project GENIE, which may limit the generalizability of genomic findings to all populations [23,24]. Notably, race- and ethnicity-stratified analyses of *FGFR4* mutations have been largely absent from the existing literature, as prior studies have primarily focused on tumor type and mutational frequency without demographic stratification [1,9,16]. Our findings highlight the importance of incorporating demographic variables into future genomic studies of *FGFR4* to determine whether population-specific differences in mutation distribution have implications for therapeutic targeting or prognostic assessment.

### 4.4. Sample Type and Tumor Site Distribution Across FGFR4 Mutation Groups

Statistically significant differences in sample type distribution were observed across FGFR4 mutation groups, though these findings should be interpreted descriptively given the limitations of registry-based data. Primary tumor samples were most prominent in the *FGFR4* MUT (401) group (61.73%), while the larger domain-based groups showed a more balanced distribution of primary and metastatic samples. This raises the possibility that certain FGFR4 mutations may be more commonly found in primary tumors, while others may be more frequently associated with metastatic disease. In breast cancer, *FGFR4* mutation rates were significantly higher in metastatic samples compared to primary tumors, with hotspot mutations at residues N535 and V550 enriched in breast cancer metastases [1]. Similarly, *FGFR4*-activating mutations in rhabdomyosarcoma have been shown to promote invasiveness and metastatic spread in xenograft models [25]. *FGFR4* has also been implicated in promoting epithelial–mesenchymal transition in colorectal cancer, a process closely linked to metastatic progression [15].

The observed difference in distant organ metastasis samples in the FGFR4 MUT (401) group compared to the other groups is noted descriptively. This warrants further investigation, particularly given that residue 401 has not been well characterized in the existing FGFR4 literature. Whether this enrichment reflects a biological role for this alteration in promoting distant spread or is an artifact of the cancer types represented in this group cannot be determined from these data alone. Notably, a substantial proportion of samples lacked detailed sample type annotation, classified as “Not Collected” or “Not otherwise specified,” particularly in the *FGFR4* MUT (256–350) and *FGFR4* MUT (467–743) groups. This incomplete annotation limits the ability to draw definitive conclusions about the relationship between specific *FGFR4* mutation domains and disease stage and highlights a broader limitation of registry-based genomic databases [23].

### 4.5. Mutation Count Across FGFR4 Mutation Groups

Statistically significant differences in mutation counts were observed across FGFR4 mutation groups. These differences may reflect variation in the cancer types represented within each group and should be interpreted descriptively. When a tumor is driven by a single strong oncogenic alteration, such as an FGFR fusion or activating mutation, it tends to accumulate fewer additional mutations overall, and this is known as oncogene addiction [26]. This has been observed in FGFR3-altered bladder cancers, where tumors with FGFR3 mutations had fewer total genomic alterations than those without [26]. Whether a similar phenomenon applies to our study cannot be fully determined from this analysis. In our cohort, the *FGFR4* MUT (161–236) and *FGFR4* MUT (256–350) groups exhibited relatively low mutation counts (medians of 17 and 16, respectively), though the biological basis for these differences cannot be determined from this analysis. The elevated mean mutation count in the FGFR4 MUT (401) group (398.43) is largely attributable to a small number of outlier samples, as reflected by the substantially lower median of 22. This discrepancy indicates that the typical tumor in this group does not carry an unusually high mutation burden, and conclusions about genomic instability in this group are not supported by these data alone [18]. The *FGFR4*MUT (467–743) group, which covers the kinase domain, showed that it was similarly influenced by a small number of highly mutated samples, possibly due to the wide variety of cancer types within this group [9,16]. Overall, these findings indicate that mutation counts vary across FGFR4 mutation groups in a descriptive sense. The clinical implications of these differences, including any potential relevance to immunotherapy response, cannot be determined from this observational analysis alone [14].

### 4.6. Co-Occurring Genomic Alterations Across FGFR4 Mutation Groups

A higher frequency of co-occurring mutations was observed in the FGFR4 MUT (401) group compared to the domain-based groups; however, sensitivity analyses indicated that this apparent enrichment did not remain significant after excluding hypermutated outliers or adjusting for mutation count, cancer type, sample type, and sequencing panel. We therefore interpret these co-alteration findings descriptively, as the apparent enrichment is likely influenced by a subset of hypermutated tumors rather than reflecting a mutation-specific biological relationship between FGFR4 MUT (401) and these co-occurring genes. Co-alterations in chromatin remodeling genes including ARID1A, CREBBP, and KMT2C were among those observed at higher frequencies in this group and their loss may be linked to impaired DNA repair; however, whether this phenomenon applies to our study cannot be fully established from this analysis [27]. The co-occurrence of alterations in chromatin remodeling and DNA damage repair genes in the MUT (401) group is noted, though whether these associations reflect any mechanistic relationship cannot be established from these observational data [18,27]. The co-occurrence of tumor suppressor mutations in APC and NF1 and receptor tyrosine kinase alterations in ALK, ERBB4, and ROS1 were also observed. However, no causal or functional relationship between these co-alterations and FGFR4 MUT (401) can be inferred from these data [16]. These patterns of co-occurrence have not been previously described for *FGFR4* and highlight the importance of considering the broader mutational context when evaluating *FGFR4* as a therapeutic target [3,14].

### 4.7. Driver vs. Passenger Status of FGFR4 Mutations

The majority of FGFR4 mutations in this cohort (6127) are classified as variants of unknown significance, with only 134 confirmed as drivers. These driver mutations were in the kinase domain—N535K/D and V550L/M/E/G. The N535K/D is a known oncogenic alteration in breast cancer that promotes ligand-independent receptor activation and downstream MAPK signaling and PI3K/AKT [1]. The V550E has also been shown to drive tumor invasiveness and metastasis in rhabdomyosarcoma [20]. All the confirmed drivers cluster in the kinase domain rather than extracellular domains which support the gain-of-function alterations that are likely to drive cancer growth. These residues may therefore represent the most promising candidates for targeting with selective FGFR4 inhibitors [14,16]. However, the overwhelming majority of FGFR4 mutations, including those at residue 401, remain functionally uncharacterized and may represent either low-penetrance drivers or passenger events. Future functional studies will be needed to determine which of these variants have true oncogenic potential.

We did not apply functional impact prediction tools such as SIFT, PolyPhen, or CHASM, nor did we assess clonal architecture or mutation timing. While OncoKB annotations confirmed 134 FGFR4 driver mutations, the vast majority remain classified as variants of unknown significance, highlighting the need for further functional characterization.

### 4.8. Validation Analysis

**Figure 8:**
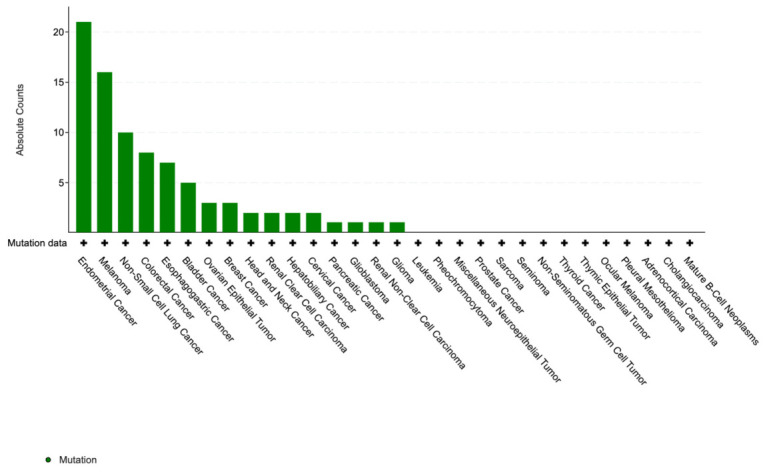
TCGA Cancer Type Distribution.

**Figure 9:**
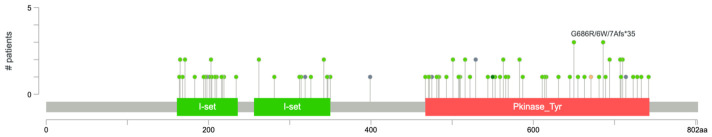
TCGA Mutation Domain Distribution (Lollipop Plot).

**Figure 10:**
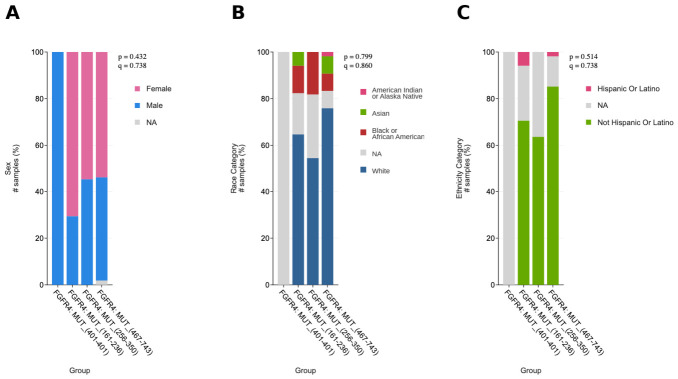
TCGA Demographic Distribution. (**A**) Sex Distribution. (**B**) Race Distribution. (**C**) Ethnicity Distribution.

To confirm the robustness of our findings, we performed a parallel analysis in the TCGA PanCancer Atlas cohort, which included 85 patients with FGFR4 alterations. Several findings from the primary GENIE analysis were replicated in this cohort. FGFR4 alterations were distributed across a broad range of cancer types, with endometrial cancer, melanoma, and non-small cell lung cancer among the most frequently represented in Figure 8, consistent with the pan-cancer pattern observed in GENIE. The domain-level distribution of mutations was similarly conserved, with alterations clustering within the immunoglobulin I-set and protein tyrosine kinase domains shown in Figure 9. Sex did not differ significantly across mutation groups (*p* = 0.432, q = 0.738), consistent with the null finding in the GENIE cohort shown in Figure 10A.

**Figure 11:**
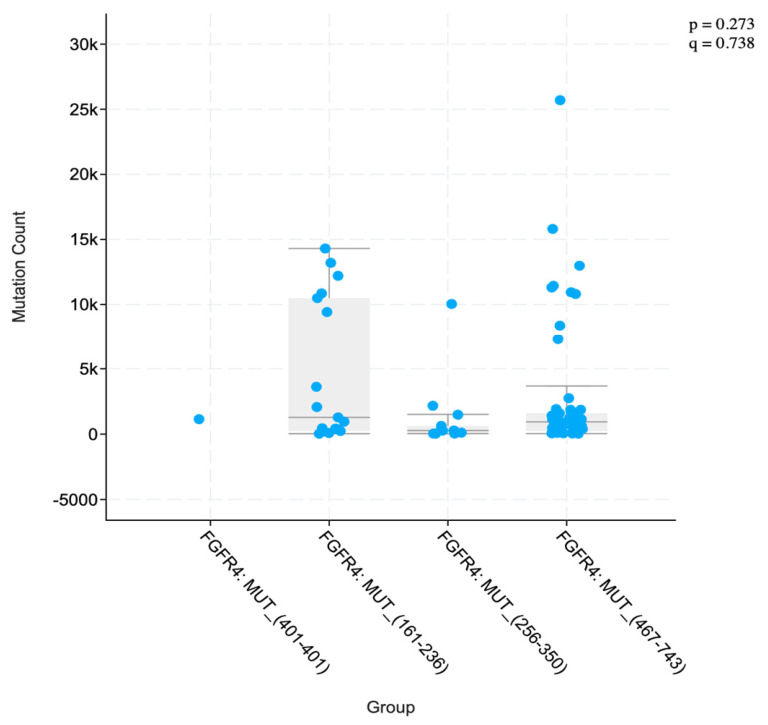
TCGA Mutation Count Distribution.

Several findings from the primary analysis were not replicated in the TCGA cohort. The statistically significant differences in racial and ethnic distribution observed in GENIE (*p* = 2.300 × 10^−6^ and *p* = 8.332 × 10^−4^, respectively) were not present in TCGA (*p* = 0.799 and *p* = 0.514, respectively) shown in Figure 10B,C. Mutation counts also did not differ significantly across groups in TCGA (*p* = 0.273, q = 0.738) shown in Figure 11, in contrast to the significant differences observed in GENIE (*p* = 2.084 × 10^−3^, q = 4.630 × 10^−3^). These discrepancies are most likely attributable to the substantially smaller sample size of the TCGA cohort which was nearly 100 times smaller, and particularly the FGFR4 MUT (401) group, which contained only one sample, precluding meaningful group-level comparisons. These results should therefore be interpreted as a limited exploratory comparison rather than a formal validation, and larger independent datasets will be needed to confirm the patterns observed in the primary analysis.

### 4.9. Strengths and Limitations

This study has several strengths. It uses one of the largest publicly available cancer genomic databases, the AACR Project GENIE, with 4565 FGFR4-mutated tumor samples across many cancer types. This pan-cancer approach provides a broader view of FGFR4 than prior studies, which have mostly focused on single cancers such as hepatocellular carcinoma or breast cancer. Our domain-level analysis of FGFR4 mutations is also a new contribution, as previous studies have mainly looked at individual variants like p.Gly388Arg rather than mapping mutations across functional regions of the protein. Additionally, by including analyses of demographics, sample type, mutation burden, and co-occurring alterations, this study offers a more complete picture of FGFR4 than what has been previously reported. The identification of hotspot 401 as a subgroup with elevated mutation burden—and the use of sensitivity analyses to test whether its co-alteration pattern reflects a real biological association—is another novel contribution that may guide future research.

This study also has limitations. The AACR GENIE database includes data from multiple institutions using different sequencing platforms and gene panels, which may lead to differences in how mutations are detected and reported. The cohort also reflects the demographic makeup of the contributing institutions, where racial and ethnic minorities are known to be underrepresented [23,24]. A large portion of race, ethnicity, and sample type data was missing or listed as “Unknown” or “Not Collected,” which limits the ability to detect meaningful patterns. While a parallel analysis in the TCGA PanCancer Atlas cohort was performed, the limited sample size of that cohort precluded formal independent validation, and experimental functional validation was beyond the scope of this study.

## 5. Conclusions

*FGFR4* alterations were identified across a broad range of malignancies, with non-small cell lung cancer, colorectal cancer, and melanoma representing the most frequently observed tumor types. This supports *FGFR4* as a recurrent altered gene across multiple cancer types. These alterations clustered predominantly within the protein tyrosine kinase domain and the immunoglobulin I-set domains. Their overall distribution did not vary significantly across cancer types. Sex was not significantly associated with the *FGFR4* mutation group, suggesting that it is unlikely to be a major determinant of *FGFR4* mutational patterns. In contrast, race and ethnicity differed significantly across mutation groups. White patients comprised the majority across groups. Asian patients had a higher frequency of *FGFR4* MUT (401) and *FGFR4* MUT (161–236), and Spanish/Hispanic patients had an increased frequency in *FGFR4* MUT (401). However, these findings should be interpreted cautiously, given the demographic composition of the contributing institutions to the AACR Project GENIE.

Differences in sample type distribution were also observed, with primary tumors more prominently represented in the FGFR4 MUT (401) group compared to the domain-based groups. All of the other larger domain-based groups demonstrated a more even distribution of primary and metastatic samples. It is also important to note that incomplete sample type annotation across the cohort remains a limiting factor in drawing definitive conclusions. Similarly, mutation burden differed across groups, with *FGFR4* MUT (401) showing the highest mean mutation count, which was largely driven by a subset of highly mutated tumors. The domain-based groups had far lower mutation counts overall. In addition, co-occurring alterations in chromatin remodeling, DNA damage repair, tumor suppressor, and receptor tyrosine kinase genes were observed at higher frequencies in the FGFR4 MUT (401) group; however, sensitivity analyses indicated that this apparent enrichment did not persist after excluding hypermutated outliers or adjusting for mutation count, cancer type, sample type, and sequencing panel. These are therefore descriptive findings likely attributable to a subset of hypermutated tumors, and no causal or functional relationships can be inferred from this analysis. A parallel analysis in the TCGA PanCancer Atlas cohort was performed; however, the limited sample size of that cohort, particularly the FGFR4 MUT (401) group which contained only one sample, precluded formal independent validation. Larger external datasets will be needed to confirm the patterns observed in this study. Taken together, these results provide a broader picture of *FGFR4* alterations across cancer types and support continued study of *FGFR4* in cancer biology.

## Data Availability

The data presented in this study are available in the AACR Project GENIE repository at https://genie.cbioportal.org/. These data were derived from the following public domain resource: AACR Project GENIE via cBioPortal (https://genie.cbioportal.org/), accessed on 12 April 2026.

## Supplementary Materials

The following supporting information can be downloaded at: https://www.mdpi.com/article/10.3390/cimb48070748/s1.

## Abbreviations

The following abbreviations are used in this manuscript:

FGFR4	Fibroblast Growth Factor Receptor 4
FGFR1–3	Fibroblast Growth Factor Receptors 1–3
AACR	American Association for Cancer Research
GENIE	Genomics Evidence Neoplasia Information Exchange
cBioPortal	Cancer Bioinformatics Portal
TCGA	The Cancer Genome Atlas
CAN	Copy Number Alteration
NSCLC	Non-Small Cell Lung Cancer
MAPK	Mitogen-Activated Protein Kinase
PI3K/AKT	Phosphatidylinositol 3-Kinase/Protein Kinase B
SD	Standard Deviation
FDR	False Discovery Rate
ARID1A	AT-Rich Interaction Domain 1A
CREBBP	CREB Binding Protein
KMT2C	Lysine Methyltransferase 2C
APC	Adenomatous Polyposis Coli
NF1	Neurofibromin 1
ATM	Ataxia Telangiectasia Mutated
BRCA2	Breast Cancer Gene 2
POLE	DNA Polymerase Epsilon
NOTCH1	Notch Receptor 1
NOTCH3	Notch Receptor 3
ERBB4	Erb-B2 Receptor Tyrosine Kinase 4
ALK	Anaplastic Lymphoma Kinase
ROS1	ROS Proto-Oncogene 1
BRAF	B-Raf Proto-Oncogene
IRB	Institutional Review Board
