# Supplementary material for: Mutational Landscape of FGFR4 Across Malignancies: A Cross-Cancer Analysis of the AACR Project GENIE Database
Source: Curr Issues Mol Biol. 2026 Jul 22;48(7):748. doi: 10.3390/cimb48070748 (PMC13407695; doi:10.3390/cimb48070748)
Supplement: Supplementary file 1 [file cimb-48-00748-s001.zip › cimb-4369421-supplementary.pdf]

Supplementary tables

Table S1. Distribution of cancer types across FGFR4 mutation groups.

Distribution of Cancer Types Across FGFR4 Mutation Groups							
Cancer Type	FGFR4: MUT_(10-10)	FGFR4: MUT_(136-136)	FGFR4: MUT_(388-388)	FGFR4: MUT_(401-401)	FGFR4: MUT_(161-236)	FGFR4: MUT_(256-350)	FGFR4: MUT_(467-743)
**Hepatobiliary Cancer**	1	0	1	1	6	6	24
**Non-Small Cell Lung Cancer**	0	3	1	10	74	75	248
**Glioma**	0	1	0	11	20	28	84
**Colorectal Cancer**	0	0	1	5	37	46	223
**Cancer of Unknown Primary**	0	0	1	2	25	17	82
**Melanoma**	0	0	0	6	46	56	142
**Mature B-Cell Neoplasms**	0	0	0	3	6	4	21
**Gastrointestinal Stromal Tumor**	0	0	0	1	0	0	4
**Adrenocortical Carcinoma**	0	0	0	1	1	0	1
**Soft Tissue Sarcoma**	0	0	0	2	13	7	61
**Head and Neck Cancer**	0	0	0	2	4	6	13
**Prostate Cancer**	0	0	0	4	9	17	42
**Ovarian Cancer**	0	0	0	4	15	20	44
**CNS Cancer**	0	0	0	1	3	1	10
**Breast Cancer**	0	0	0	3	41	39	134
**Germ Cell Tumor**	0	0	0	1	0	1	1
**Penile Cancer**	0	0	0	1	0	0	1
**Renal Cell Carcinoma**	0	0	0	3	6	5	14
**Peritoneal Cancer, NOS**	0	0	0	1	1	0	0
**Skin Cancer, Non-Melanoma**	0	0	0	2	6	11	31

Non-overlapping sample counts are shown for each cancer type stratified by mutation group, including hotspot mutations at amino acids 10, 136, 388, and 401, and domain-level mutations within the immunoglobulin I-set domains (161–236 and 256–350) and the protein tyrosine kinase domain (467–743). Hotspots at amino acids 10, 136, and 388 were excluded from further subgroup analyses due to limited sample sizes (n≤4). The total denominator for percentage calculations was 2,809 non-overlapping samples.

Table S2. Summary statistics of mutation counts across *FGFR4* mutation groups.

Summary Statistics of Mutation Counts Across FGFR4 Mutation Groups				
**Statistic**	**FGFR4: MUT_(401-401)**	**FGFR4: MUT_(161-236)**	**FGFR4: MUT_(256-350)**	**FGFR4: MUT_(467-743)**
**Count**	81	396	425	1580
**Minimum**	2	1	1	1
**Maximum**	3363	621	638	4441
**Mean**	398.43	45.67	42.04	97.4
**Standard Deviation**	659.68	80.36	71.1	264.65
**Median**	22	17	16	19
**Mean Absolute Deviation**	16	11	9	13
**25% (Q1)**	10	10	9	9
**75% (Q3)**	877	49	45	68

Table S3. Co-occurring Genomic Alterations Across *FGFR4* Mutation Groups.

Co-occurring Genomic Alterations Across FGFR4 Mutation Groups								
**Gene**	**Cytoband**	**FGFR4: MUT_(401-401)**	**FGFR4: MUT_(161-236)**	**FGFR4: MUT_(256-350)**	**FGFR4: MUT_(467-743)**	**p-Value**	**q-Value**	**Most Enriched In**
**ARID1A**	1p36.11	31 (38.27%)	84 (21.37%)	96 (22.86%)	496 (31.82%)	3.226e-6	1.465e-5	**FGFR4: MUT_(401-401)**
**APC**	5q22.2	33 (40.74%)	73 (18.58%)	86 (20.33%)	442 (28.06%)	8.19e-7	4.052e-6	**FGFR4: MUT_(401-401)**
**NF1**	17q11.2	30 (37.04%)	81 (20.51%)	95 (22.35%)	441 (28.14%)	4.729e-4	1.640e-3	**FGFR4: MUT_(401-401)**
**ATM**	11q22.3	29 (35.80%)	69 (17.47%)	80 (18.87%)	416 (26.40%)	7.442e-6	3.205e-5	**FGFR4: MUT_(401-401)**
**BRCA2**	13q13.1	34 (41.98%)	78 (19.75%)	74 (17.45%)	390 (24.90%)	2.339e-6	1.080e-5	**FGFR4: MUT_(401-401)**
**CREBBP**	16p13.3	34 (41.98%)	67 (17.05%)	73 (17.38%)	378 (24.29%)	2.57e-7	1.336e-6	**FGFR4: MUT_(401-401)**
**ROS1**	6q22.1	34 (41.98%)	85 (21.52%)	78 (18.40%)	374 (23.90%)	4.908e-5	1.974e-4	**FGFR4: MUT_(401-401)**
**NOTCH3**	19p13.12	33 (41.77%)	79 (22.13%)	61 (16.44%)	373 (26.12%)	2.320e-6	1.076e-5	**FGFR4: MUT_(401-401)**
**KMT2C**	7q36.1	38 (47.62%)	67 (26.27%)	70 (24.73%)	370 (36.49%)	1.220e-5	5.137e-5	**FGFR4: MUT_(401-401)**
**KMT2A**	11q23.3	20 (24.69%)	67 (17.18%)	72 (17.22%)	361 (23.08%)	8.746e-3	0.0253	**FGFR4: MUT_(401-401)**
**NOTCH1**	9q34.3	34 (41.98%)	81 (20.45%)	71 (16.71%)	357 (22.59%)	6.679e-6	2.914e-5	**FGFR4: MUT_(401-401)**

Table S4. Detailed characteristics of FGFR4 Driver Mutations.

Detailed Characteristics of FGFR4 Driver Mutations							
Sample ID	Cancer Type (Detailed)	Protein Change	Annotation	Mutation Type	Copy #	Allele Freq (T)	# Mut in Sample
**GENIE-MSK-P-0026288-…**	Invasive Breast Carcinoma	FGFR4-CDK12 Fusion	Fusion	Fusion	Diploid	—	6
**GENIE-DFCI-028707-290…**	Invasive Breast Carcinoma	N535K	Oncogenic	Missense	Gain	0.28	15
**GENIE-DFCI-111495-617…**	Embryonal Rhabdomyosarcoma	N535K	Oncogenic	Missense	Diploid	0.53	8
**GENIE-DFCI-237571-330…**	Adenocarcinoma, NOS	N535K	Oncogenic	Missense	Diploid	0.4	12
**GENIE-DFCI-325155-677…**	Uterine Carcinosarcoma/Mixed	N535K	Oncogenic	Missense	Diploid	0.41	8
**GENIE-DFCI-449193-465…**	GI Neuroendocrine Tumors	N535K	Oncogenic	Missense	Diploid	0.33	10
**GENIE-DFCI-449936-475…**	Colorectal Adenocarcinoma	N535K	Oncogenic	Missense	Diploid	0.37	18
**GENIE-DFCI-527064-509…**	Invasive Breast Carcinoma	N535K	Oncogenic	Missense	Diploid	0.31	14
**GENIE-MSK-P-0094888-683…**	Embryonal Rhabdomyosarcoma	V550E	Oncogenic	Missense	Diploid	0.62	7
**GENIE-MSK-P-0003333-…**	Breast Invasive Cancer, NOS	N535K	Oncogenic	Missense	Diploid	0.34	37
**GENIE-MSK-P-0004987-…**	Breast Invasive Lobular Carcinoma	N535K	Oncogenic	Missense	Diploid	0.14	43
**GENIE-MSK-P-0006249-…**	Breast Invasive Ductal Carcinoma	N535K	Oncogenic	Missense	Diploid	0.29	20
**GENIE-MSK-P-0009602-…**	Breast Invasive Carcinoma, NOS	N535K	Oncogenic	Missense	Diploid	0.23	46
**GENIE-MSK-P-0010245-…**	Breast Invasive Lobular Carcinoma	N535K	Oncogenic	Missense	Diploid	0.29	5
**GENIE-MSK-P-0013568-…**	Breast Invasive Lobular Carcinoma	N535K	Oncogenic	Missense	Diploid	0.57	9
**GENIE-MSK-P-0028802-…**	Rectal Adenocarcinoma	N535K	Oncogenic	Missense	Diploid	0.35	10
**GENIE-MSK-P-0031802-…**	Invasive Breast Carcinoma	N535K	Oncogenic	Missense	Diploid	0.31	6
**GENIE-MSK-P-0035024-…**	Invasive Breast Carcinoma	N535K	Oncogenic	Missense	Diploid	0.26	6
